# Management of Class III Malocclusion with Microimplant-Assisted Rapid Palatal Expansion (MARPE) and Mandible Backward Rotation (MBR): A Case Report

**DOI:** 10.3390/medicina60101588

**Published:** 2024-09-27

**Authors:** Heng-Ming Chang, Chao-Tzu Huang, Chih-Wei Wang, Kai-Long Wang, Shun-Chu Hsieh, Kwok-Hing Ho, Yu-Jung Liu

**Affiliations:** 1 Orthodontic and Dental Department, Chang Bing Show Chwan Memorial Hospital, Changhua 505, Taiwan; 2 Orthodontic Department, Chang Bing Show Chwan Memorial Hospital, Changhua 505, Taiwan; hiimdianehuang@gmail.com (C.-T.H.); chihwei0114@msn.com (C.-W.W.); chordater@hotmail.com (K.-L.W.); markortho@yahoo.com.tw (S.-C.H.); yatpong@gmail.com (K.-H.H.); 3 Dental Department, Show Chwan Memorial Hospital, Changhua 500, Taiwan; jerrybon1@hotmail.com

**Keywords:** Class III malocclusion, microimplant-assisted rapid palatal expansion, mandibular backward rotation

## Abstract

Class III malocclusion prevalence varies significantly among racial groups, with the highest prevalence observed in southeast Asian populations at 15.80%. These malocclusions often involve maxillary retrognathism, mandibular prognathism, or both, accompanied by maxillary constriction and crossbites. Comprehensive treatment should address anteroposterior, transverse, and vertical imbalances. Microimplant-assisted rapid palatal expansion (MARPE) has shown high success rates for transverse maxillary expansion in late adolescents and adults, presenting a viable alternative to surgically-assisted rapid palatal expansion (SARPE). This case report aims to demonstrate the successful treatment of a young adult female with borderline Class III malocclusion using MARPE and mandibular backward rotation (MBR) techniques. A 21-year-old female presented with a Class III skeletal pattern, anterior/posterior crossbites, and mild dental crowding. Despite her concerns about a concave facial profile, the patient declined orthognathic surgery due to a negative experience reported by a friend. The treatment plan included MARPE to correct maxillary transverse deficiency and MBR to alleviate Class III malocclusion severity. Lower arch distalization was performed using temporary anchorage devices (TADs) on the buccal shelves, and Class II elastics were used to maintain MBR and prevent retroclination of the lower labial segment during anterior retraction. Significant transverse correction was achieved, and the severity of Class III malocclusion was reduced. The lower dentition was effectively retracted, and the application of Class II elastics helped maintain MBR. The patient’s final facial profile was harmonious, with well-aligned dentition and a stable occlusal relationship. The treatment results were well-maintained after one year. The MARPE with MBR approach presents a promising alternative for treating borderline Class III cases, particularly for patients reluctant to undergo orthognathic surgery. This case report highlights the effectiveness of combining MARPE and MBR techniques in achieving stable and satisfactory outcomes in the treatment of Class III malocclusion.

## 1. Introduction

Class III malocclusion prevalence varies significantly among racial groups, ranging from 1.17% to 26.67%, with an average prevalence of 7.04% [1]. Southeast Asian populations exhibit the highest prevalence at 15.80%. Class III malocclusions typically manifest as maxillary retrognathism, mandibular prognathism, or a combination of both [2], frequently accompanied by maxillary constriction and anterior and posterior crossbites. Thus, comprehensive treatment of Class III malocclusion should address not only the anteroposterior relationship but also the transverse and vertical imbalances [3].

Recent studies have highlighted microimplant-assisted rapid palatal expansion (MARPE) as an effective method for expanding the midpalatal and maxillary sutures in late adolescence and young adulthood [4,5,6]. A systematic review reported a high success rate of 92.5% for transverse maxillary expansion with MARPE, with success rates ranging from 80.65% to 100% [7]. In this article, the author defined the success of treatment as the opening of the midpalatal suture and the achievement of the required maxillary width. They concluded that the mean skeletal expansion component accounted for 35.6% of the total dental expansion. Therefore, MARPE presents a promising alternative for late adolescents and adults who are not ideal candidates for surgically-assisted rapid palatal expansion (SARPE) or more complex orthognathic surgical procedures involving multiple segments [8,9].

Liao et al. [6] emphasized the skeletal benefits of maxillary advancement and mandibular posterior rotation achieved through MARPE, which can effectively correct Class III malocclusions. Several studies have corroborated these findings, showing increases in SNA (maxillary position) and decreases in SNB (mandibular position) following MARPE treatment [10,11]. These combined effects make MARPE particularly advantageous in the camouflage treatment of mild skeletal Class III cases with hypo- or hyper-divergent patterns.

The decision of whether a patient requires orthognathic surgery or camouflage treatment alone to correct Class III malocclusion is a complex and multifaceted issue within orthodontics. Kerr et al. [12] proposed a “rule-of-thumb” guideline to differentiate between treatment approaches. They identified four cephalometric measurements—ANB < 4°, lower incisor inclination < 83°, maxillary to mandibular length ratio < 0.84, and Holdaway angle < 3.5°—suggesting that orthognathic surgery may be more appropriate than camouflage treatment alone. However, a recent systematic review highlighted the absence of a definitive guideline for orthodontists in determining the appropriate treatment (either orthognathic surgery or camouflage treatment) for borderline Class III cases [13]. The authors emphasized the urgent need for further studies employing standardized methodologies. Currently, the treatment approach for borderline Class III cases largely depends on patient expectations and the experience of the orthodontist.

## 2. Case Report

### 2.1. Diagnosis and Aetiology

A 21-year-old female patient presented with a skeletal Class III malocclusion, characterized by an average maxillo-mandibular plane angle (MMPA) of 28° (normal range: 27° ± 5°). She exhibited both anterior and posterior crossbites, along with mild dental crowding in both arches. The patient’s primary concerns were her concave facial profile and anterior crossbite. As a dental professional, she was well-versed in aesthetic reference lines, including the Esthetic Line, and expressed a desire for improved facial harmony. Despite her concerns, she declined orthognathic surgery due to a negative experience shared by a friend. The patient’s medical history was unremarkable, with no known allergies.

**Figure 1:**
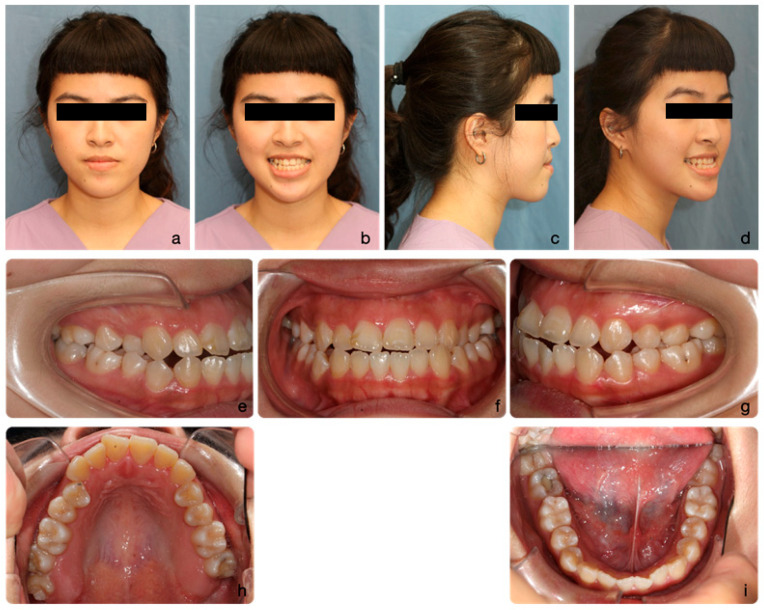
The initial facial portraits and intraoral photographs show the patient with a mildly concave profile, an average Frankfort-mandibular plane angle, and mandibular prognathism (**a**–**d**). Intraoral images reveal a Class III malocclusion with reverse overjet and anterior open bite (**e**–**g**). The maxilla appears relatively constricted in comparison to the mandible, with mild crowding observed in both arches (**h**,**i**).

On extra-oral examination, she exhibited a mild concave profile with mandibular prognathism relative to the zero meridian line. Her upper lip was retrusive (−3 mm to the E-line) and her midface lacked fullness over the malar area, presenting a flat appearance. Vertical facial proportions were balanced, albeit with slightly increased lower facial height. Her upper incisors were prominently displayed. Mild facial asymmetry was noted from the frontal view, with the chin deviated to the left (Figure 1a–d).

Intraoral examination revealed mild crowding in both the upper (3 mm) and lower (2 mm) arches. The maxilla appeared relatively constricted compared to the mandible. Occlusally, both canines and molars exhibited Class III relationships (Angle classification) bilaterally. The incisor relationship was also Class III (British Standard Institute, 1983), with a negative overjet (−2 mm) and a slight anterior open bite. The upper dental midline coincided with the facial midline, while the lower dental midline deviated 2 mm to the left. Buccal segment crossbites were observed bilaterally over the premolar area (Figure 1e–i).

**Figure 2:**
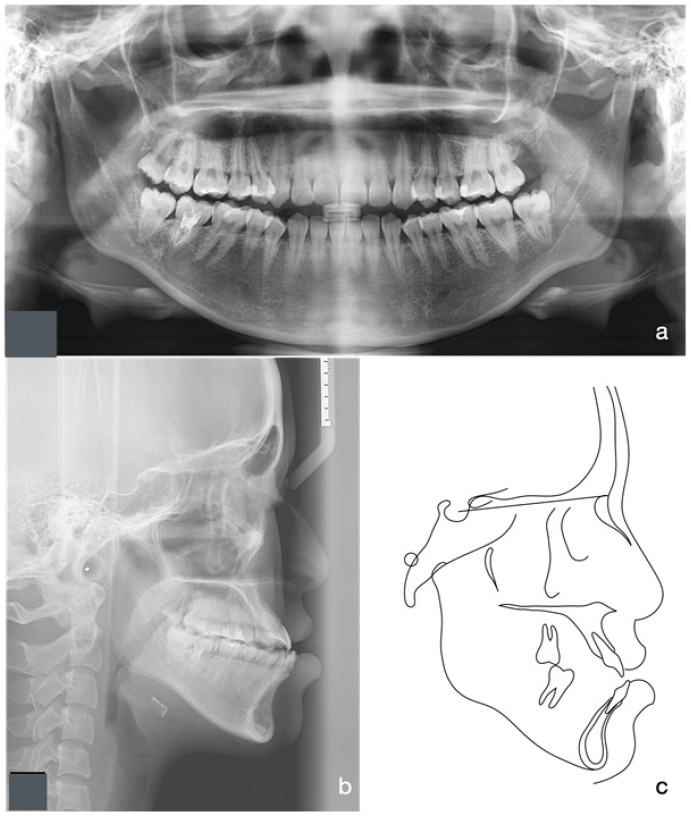
Initial Orthopantomographic (OPG) and lateral cephalometric radiographs and tracing. Orthopantomographic (OPG) radiographic examination revealed no significant caries or pathological conditions, and the patient exhibited good periodontal status. The lower right second molar had undergone endodontic treatment with no apical lesion present (**a**). Cephalometric radiograph and tracing showed a skeletal Class III relationship with an average Frankfort-mandibular plane angle (**b**,**c**).

Orthopantomographic (OPG) radiographic examination revealed no significant caries or pathological conditions, and the patient exhibited good periodontal status (Figure 2a). The lower right second molar had undergone endodontic treatment with no apical lesion present. The patient also reported occasional flare-ups of pericoronitis around the bilateral lower third molars, attributed to their close proximity to the ascending ramus. Cephalometric radiograph and tracing showed a skeletal Class III relationship with an average Frankfort-mandibular plane angle (Figure 2b,c). The lower labial segment showed normal inclination.

### 2.2. Treatment Objectives

The main goals of the treatment were to (1) correct the anterior and posterior crossbites, (2) address the transverse discrepancy, (3) reduce chin prominence, (4) relieve dental crowding in both arches, (5) establish Class I canine and molar relationships, and (6) improve dental and facial esthetics through non-orthognathic surgical methods.

### 2.3. Treatment Alternatives

Orthognathic surgery combined with orthodontic treatment is the first choice of treatment for this patient. However, after a thorough discussion, the patient declined this option due to her friend’s unpleasant experience and requested orthodontic camouflage treatment alone. The patient’s cephalometric measurements of ANB, lower incisor inclination, the ratio of maxillary to mandibular lengths, and Holdaway angle are −2.5°, 94°, 0.69 (77 mm/112 mm), and 7.9°, respectively. Two out of these four measures indicate suitability for camouflage treatment according to Kerr et al. [12] However, the low maxillo-mandibular length ratio and increased Holdaway angle require careful management. Given the patient’s slight maxillary retrusion, maxillary advancement could improve the maxillo-mandibular length ratio. Additionally, distalization and retraction of the lower anterior teeth could improve the increased Holdaway angle.

Due to the patient’s age and preference, MARPE was chosen to correct the transverse problem instead of conventional RPE or SARPE. Regarding lower anterior teeth distalization and retraction, the patient presented only mild crowding in both arches, and the initial reverse overjet was only 2 mm. We decided to extract four third molars instead of four premolars for anterior distalization. We aimed to distalize the lower dentition with temporary anchorage devices (TADs) on both sides of the buccal shelves. The decision to extract the lower right third molar, rather than the endodontically treated second molar, was due to the absence of keratinized gingiva on the buccal side of the third molar and its occasional pericoronitis flare-ups. The lower right second molar had intact crown walls, and the endodontic treatment was repeated before initiating orthodontic treatment.

**Figure 3:**
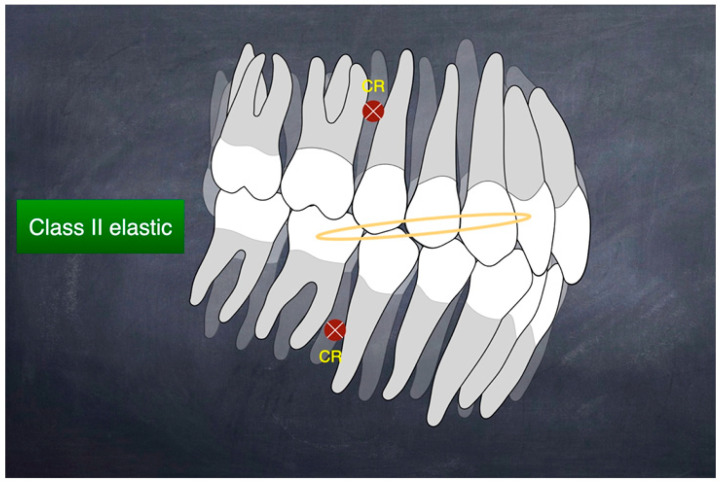
Treatment mechanics of Class II elastic (yellow ellipse) causing occlusal plane backward rotation. Class II elastics can simultaneously promote extrusion of the lower molars and upper incisors, resulting in occlusal plane backward rotation. This effect improves upper incisor display and helps maintain the backward rotation of the mandible.

During the lower arch distalization period, the lower posterior teeth would inevitably be intruded and the anterior teeth extruded [14]. This could cause posterior bite opening and anterior bite deepening, leading to forward rotation of the mandible. To maintain the mandibular backward rotation (MBR) achieved by MARPE, Class II elastics were used to maintain the molar vertical position, and, simultaneously, the upper anterior teeth could be extruded. This approach could lead to a backward rotation of the occlusal plane and increase the patient’s upper incisor show (Figure 3). By applying Class II elastics, the mandibular plane can be rotated backward, and the lower facial height can be slightly increased [15].

### 2.4. Treatment Progress

The patient came for an initial consultation in August 2019. The treatment progress can be roughly grouped into four stages: the MARPE stage, the initial alignment stage, anterior retraction with MBR, and the finishing stage.

**Figure 4:**
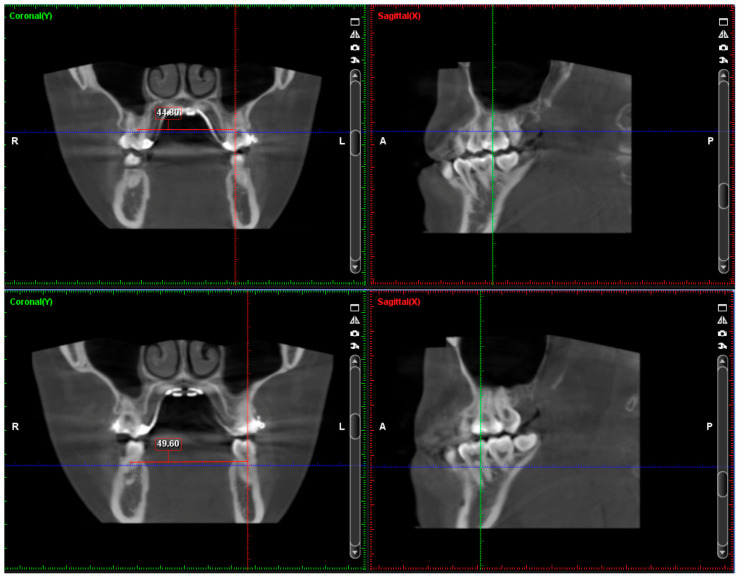
Pretreatment cone beam computed tomography (CBCT) images and measures. Inter-furcation distance of upper first molars was 44.8 mm, and inter-furcation distance of lower first molars was 49.6 mm. According to Yonsei Transverse Index (YTI), about 5 mm of maxillary expansion was needed. (Red line: Sagittal plane; green line: Coronal plane; blue line: Axial plane).

**Figure 5:**
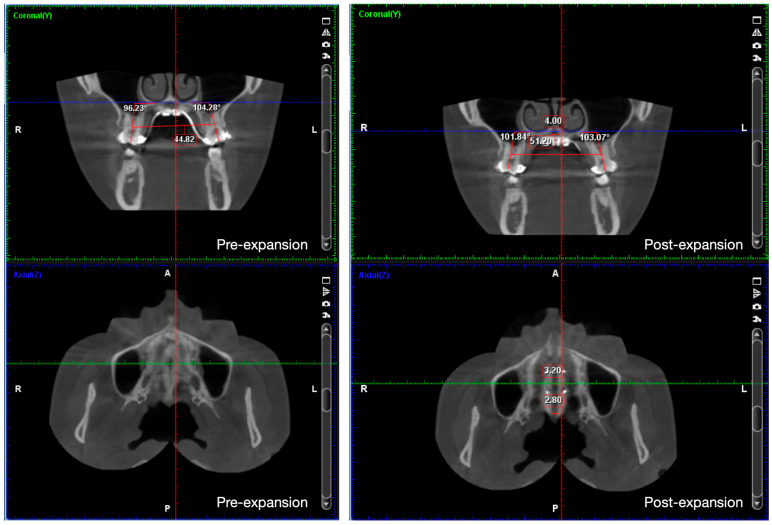
Pre- and post-expansion CBCT images and measures. Molar inclinations were not worsened after MARPE. Expansion pattern was fairly parallel from anterior to posterior on the axial view. (Red line: Sagittal plane; green line: Coronal plane; blue line: Axial plane).

**Figure 6:**
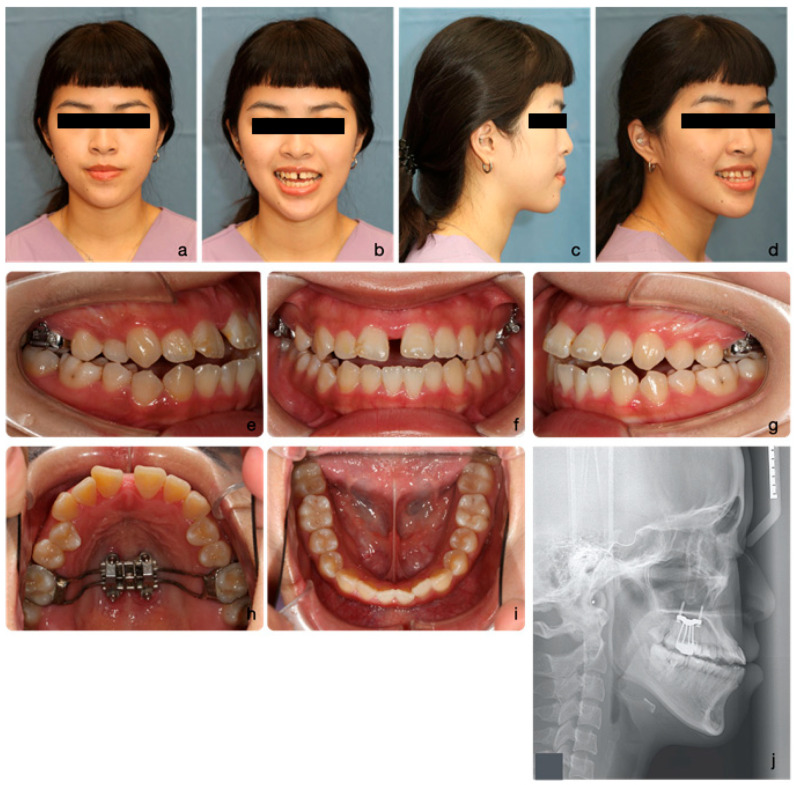
Post-MARPE facial portraits (**a**–**d**), intra-oral photos (**e**–**i**) and lateral cephalometric radiograph (**j**). A median diastema was present as a result of mid-palatal suture opening. The mandible underwent backward rotation due to changes in molar occlusion.

During the MARPE stage, a maxillary skeletal expander (MSE; BioMaterials Korea, Inc., Seoul, Republic of Korea) type-2 was used. It was positioned along the midpalatal suture at the level of the upper first molars. Four microimplants (1.8 mm in diameter; 11 mm in length) were inserted, achieving bicortical penetration, which is beneficial for anchorage support and transmitting force to the sutures around the maxilla [16]. Initial cone beam computed tomography (CBCT) showed that the patient needed around 5 mm of expansion according to the Yonsei Transverse Index (YTI) (Figure 4) [17]. After a 2-week healing period, maxillary expansion was commenced with three turns per day for 10 days until the posterior crossbites were corrected. Images from CBCT demonstrated that the midpalatal suture was successfully opened with very little buccal tipping of the posterior teeth (Figure 5). A fairly parallel expansion pattern from ANS to PNS was achieved in this patient, with amounts of 3.2 mm in the anterior and 2.8 mm in the posterior, respectively. The post-expansion photos and radiographs presented an anterior crossbite correction, resulting from maxillary advancement and MBR (Figure 6).

After three months of retention, fixed appliance treatment was commenced. During the initial alignment stage, self-ligating brackets with 0.022-inch preadjusted brackets (MBT prescription) were bonded in January 2020. The arch wire sequence used was 0.014-inch heat-activated nickel titanium (HANT), 0.018X0.025-inch HANT, and 0.019X0.025-inch stainless steel (SS) wire, respectively. After five months of alignment, four TADs (SH-1312-10, AbsoAnchor^®^, Dentos Inc., Daegu, Republic of Korea) were inserted for lower arch distalization in June 2020.

**Figure 7:**
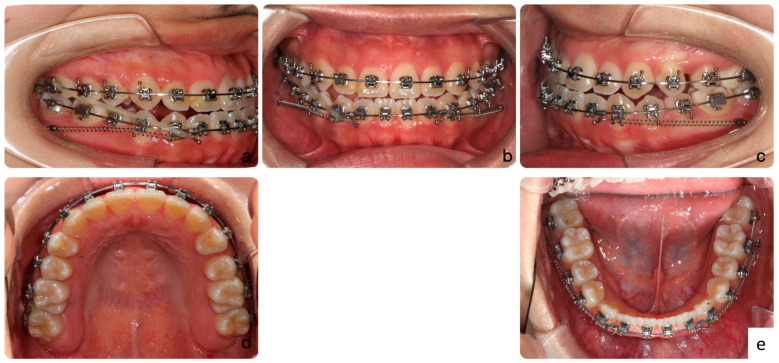
Intra-oral photos (**a**–**e**) of anterior retraction stage. During this period, a mild reverse curve of Spee was added to prevent further retroclination of the lower incisors during lower arch distalization.

In the anterior retraction with MBR stage, we aimed to reduce the anterior crossbite produced during initial alignment. NiTi coil springs were attached from the TADs to anterior hooks for lower anterior retraction. During this period, a mild reverse curve of Spee was added to prevent further retroclination of the lower incisors during retraction. Meanwhile, a right-side Class III elastic was used for lower centerline correction (Figure 7). It took four months to achieve a positive overjet and centerline coincidence.

**Figure 8:**
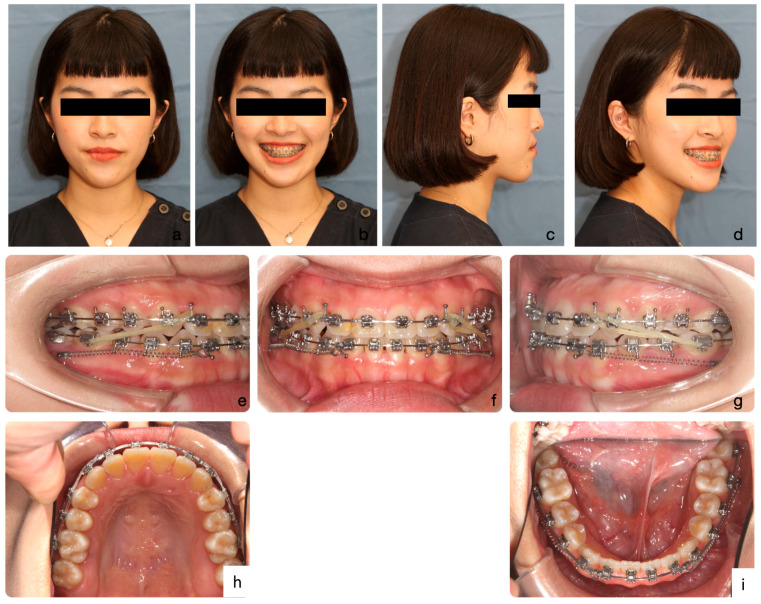
Mandible backward rotation stage (MBR) facial portraits and intra-oral photos. Facial portraits show a reduction in chin prominence and a more harmonious facial profile (**a**–**d**). Intraoral photographs demonstrate lower arch distalization using NiTi coil springs anchored by TADs, assisted by Class II elastics from the mandibular molars to the maxillary anterior hooks on both sides (**e**–**i**). This approach aimed to enhance molar extrusion and maintain the mandible in a backward position.

Since December 2020, the MBR procedure was commenced. Anterior retraction with NiTi coil springs from TADs continued, aided by Class II elastics from mandibular molars to maxillary anterior hooks on both sides (Figure 8). This approach aimed to enhance molar extrusion and maintain the mandible in a backward position. It took ten months to stabilize the extruded molars and settle the posterior occlusion.

**Figure 9:**
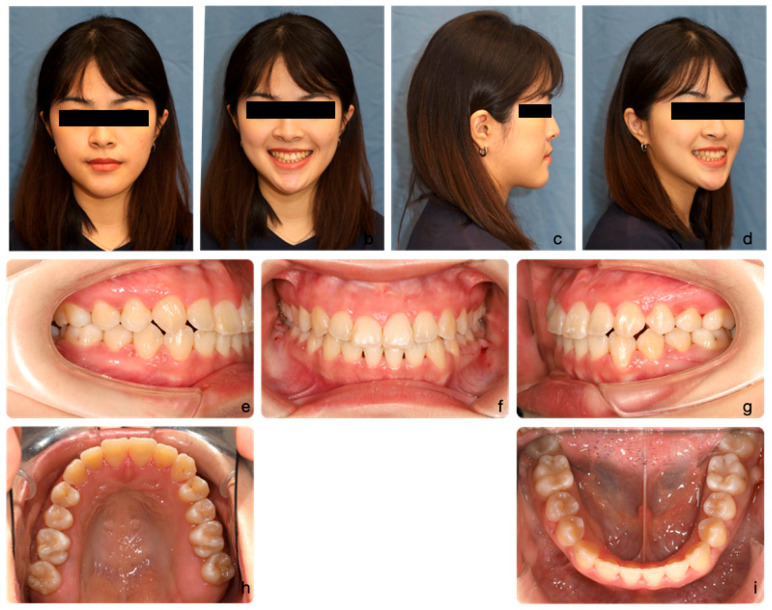
Final facial portraits and intra-oral photos. Facial portraits show a Class I skeletal pattern with a harmonious facial profile (**a**–**d**). A full-crown smile with an ideal smile arc was achieved. Intraoral photographs demonstrate good dental alignment, solid interdigitation, and proper overjet and overbite (**e**–**i**).

**Figure 10:**
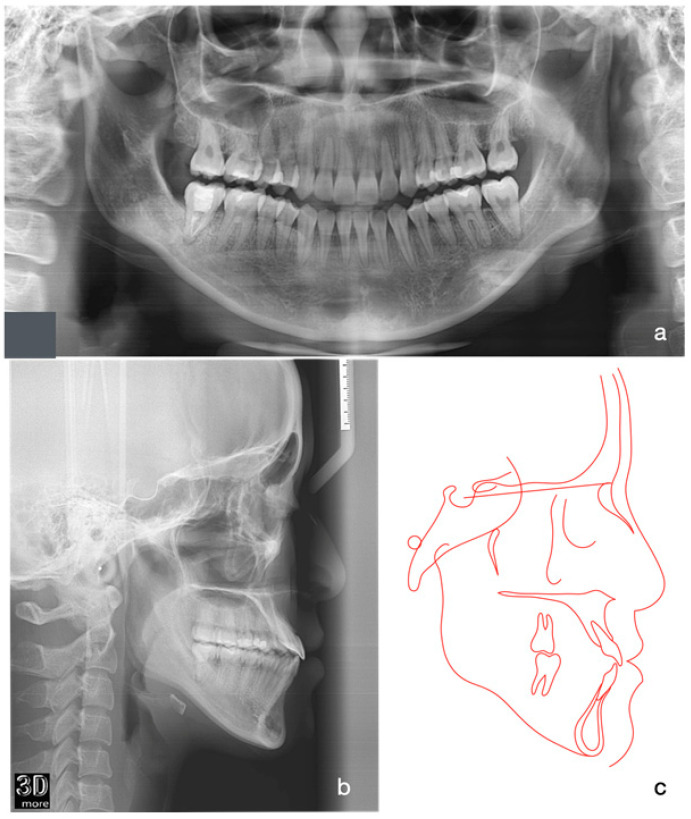
Final orthopantomographic (OPG) and lateral cephalometric radiographs and tracing. Good root parallelism was achieved, with no significant root resorption observed (**a**). Lateral cephalometric radiographs and tracings demonstrate a skeletal Class I relationship with reduced chin projection. Both the upper and lower incisors show proper inclination (**b**,**c**).

In October 2021, an orthopantomographic radiograph was taken, and several brackets were repositioned for better root parallelism. After realignment, the patient was instructed to wear bilateral box elastics to settle the occlusion over the buccal segments. After four months of finishing, the patient was debonded in March 2022, and final records were taken (Figure 9 and Figure 10). Vacuum-formed clear retainers were prescribed for retention.

### 2.5. Treatment Results

At the end of treatment, we achieved an Angle Class I molar relationship with good interdigitation and evenly distributed occlusal contacts. Both the upper and lower teeth were well aligned, and positive overjet and overbite were established. The transverse dimension of the maxillary arch improved after MARPE, and the posterior crossbites were corrected. 

The final OPG radiograph showed good root parallelism with no TMJ bony destruction or dental disease noted. Final prosthesis fabrication of the lower right second molar was recommended. 

The patient’s final facial profile was harmonious in all three dimensions, although the chin still deviated slightly to the left. The cephalometric analysis (Table 1) showed that MBR effectively reduced the severity of Class III malocclusion, with MARPE used at the start as well as Class II elastics during the lower anterior retraction stage. The mandible remained stable in its backward-rotated position, showing a 2-degree increase. This also improved the ANB angle by 2 degrees, making the Class III skeletal pattern less severe and treatable with orthodontics alone. The lower teeth were well retracted and distalized with the help of TADs on the mandibular buccal shelves. The inclinations of the upper and lower incisors were maintained without retroclinations, which is important to establish the aesthetic contour lines of both lips [18].

**Figure 11:**
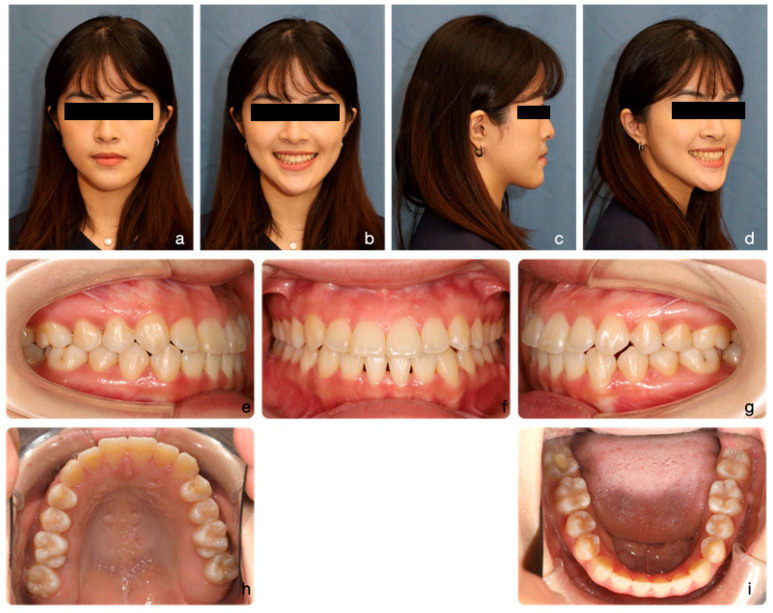
One-year-review facial portraits and intra-oral photos. The Class I skeletal pattern and harmonious facial profile were maintained (**a**–**d**). Good dental alignment, solid interdigitation, and proper overjet and overbite were also preserved (**e**–**i**).

**Figure 12:**
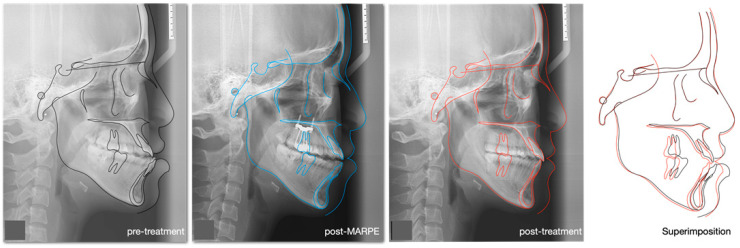
Cephalometric tracing comparisons and superimposition.

After one year of review, the patient’s facial profile and occlusion were well-maintained, with no signs of alignment relapse (Figure 11). A series of cephalometric changes and tracings are demonstrated in Figure 12. Both the mandibular and occlusal planes were rotated backward, and the upper incisors were extruded successfully.

## 3. Discussion

Class III skeletal patterns are typically associated with anterior and/or posterior crossbites. According to the recent literature, maxillary expansion using conventional rapid palatal expansion (RPE) devices in late adolescence or adulthood may result in adverse side effects, including limited skeletal effects, undesirable tooth movement, root resorption, and insufficient long-term stability [19]. SARPE is a viable alternative for adult patients; however, this procedure carries potential risks such as hemorrhage, injury to the branches of the maxillary nerve, pain, sinus infection, alar base flaring, and relapse [3].

In recent years, MARPE has demonstrated significant skeletal effects on expansion with a less invasive approach [8,9]. Our previous study reported an overall success rate of 94.7% (18/19) for midpalatal suture opening using MARPE [6]. Additionally, a recent systematic review reported a mean success rate of 92.5% (range: 80.65% to 100%), indicating that MARPE is a practical and effective alternative for maxillary expansion in adult patients with maxillary transverse deficiency.

In the current case, the anterior crossbite was spontaneously corrected following MARPE due to anterior displacement of the maxilla (SNA increased from 81° to 81.5°) and backward rotation of the mandible (SNB reduction from 83.5° to 81.5°). This combined effect rendered the borderline skeletal Class III case less severe and manageable with orthodontic treatment alone. Consistent findings have been reported in our previous study and other studies employing similar methodologies, demonstrating the same trends of maxillary anterior displacement and MBR after MARPE [6,10,11].

Shih et al. [4] demonstrated that MARPE treatment can be highly effective in late adolescents with borderline skeletal Class III patterns, average Frankfort-mandibular plane angles, and moderate dental crowding. Their study showed an increase of 1 degree in the SNA and a decrease of 1 degree in the SNB, contributing to a 2-degree improvement in the ANB after MARPE. Although the improvement in ANB angle decreased by 0.5 degrees by the end of treatment, the extent of MBR was largely maintained. The results of this case were similar to the findings of Shih et al., and therefore, MARPE should be considered a successful intervention for mild to moderate skeletal Class III cases in late adolescents and adults.

After MARPE and initial alignment, the use of TADs over the mandibular buccal shelves was effective in providing anchorage for lower arch distalization. Chen et al. [20] investigated the spatial limits during mandibular arch distalization and identified ridge width and available posterior distance as key factors determining the extent of distalization. Kim et al. [21] reported that the lingual cortex of the mandibular body is the posterior limit of molar distalization and that CBCT images can be used for better prediction.

Elshebiny et al. [22] conducted a CBCT study to identify suitable sites for orthodontic miniscrew insertion over the mandibular buccal shelf. Considering four variables—cortical bone thickness, bone width, insertion depth, and proximity to nerves—they concluded that the level of the distobuccal cusp of the mandibular second molar is the most ideal site for miniscrew insertion. Nucera et al. [23] also found that the buccal bone corresponding to the distal root of the second molar could be a suitable site for miniscrew insertion over the buccal shelf. The mandibular second molar area provides abundant space for screw insertion and preserves clearance for lower arch distalization.

MBR is beneficial for sagittal correction in Class III orthodontic treatment, provided the patient’s mandibular plane is not hyperdivergent. MBR was traditionally considered unstable and prone to relapse due to the stretch of the pterygomandibular sling (masseteric and medial pterygoid muscles). However, Class III malocclusion in adults often presents with wear on the posterior teeth due to a lack of anterior or canine guidance, leading to vertical dimension loss and increased mandibular prognathism. Tallgren et al. [24] observed this phenomenon in complete denture wearers, who exhibited upward rotation of the mandible and increased mandibular prognathism. They emphasized the importance of maintaining or restoring vertical dimensions to sustain masticatory muscle activity [25]. Applying this concept to Class III malocclusion treatment, MBR can help re-establish proper vertical dimension, which should be considered stable.

Numerous MBR techniques have been presented in the literature, all demonstrating successful results in Class III orthodontic treatment [26,27,28]. MARPE is highly effective in achieving MBR due to the establishment of new occlusal contacts after expansion. Subsequently, during the lower arch distalization stage, we applied Class II elastics to enhance the extrusion of the upper labial segment and the lower buccal segments. This approach not only maintains the MBR position after MARPE but also rotates the occlusal plane backward, increasing the upper incisor show and helping to establish a smile arc in skeletal Class III cases. The backward rotation of the occlusal plane also prevents further retroclination of the lower labial segment and deterioration of the labiomental fold. This patient presented a harmonious lateral profile with a full-crown pleasant smile at the end of treatment. The overall treatment results were well maintained after one year of review.

From the patient’s perspective, she was hesitant to undergo orthognathic surgery due to negative experiences shared by a friend. Common complications of orthognathic surgery include postoperative malocclusion, hemorrhage, inferior alveolar nerve injury, unfavorable splits, and infection [29]. Based on the guidelines proposed by Kerr et al. [12], the patient was considered a borderline case for orthognathic surgery and opted for a non-surgical treatment approach. She was aware of her mandibular prognathism and anterior crossbite and understood that MBR could reduce her chin prominence, while TADs could address the anterior crossbite. Although there was a slight increase in lower facial height at the end of treatment, the patient was satisfied with the overall result, particularly appreciating the improved curvature of her chin over the labiomental fold.

## 4. Conclusions

This case report demonstrates the successful treatment of a young adult female patient with borderline Class III malocclusion and anterior/posterior crossbites. The MARPE procedure with an MSE device not only achieved significant transverse correction but also alleviated the severity of the Class III malocclusion. The lower dentition was effectively retracted with the aid of TADs on the buccal shelves, while Class II elastics helped maintain the MBR effect and prevent retroclination of the lower labial segment during the anterior retraction stage. Therefore, the MARPE with MBR approach presents a promising alternative for borderline Class III cases in patients who are reluctant to undergo orthognathic surgery.

## Figures and Tables

**Table 1:** Cephalometric analysis comparisons among initial, post-MARPE, and post-treatment.

Sagittal Skeletal Relationships				
	Normal (SD)	Initial	Post-Expansion	Final
**SNA**	81° ± 3°	81°	81.5°	81°
**SNB**	78° ± 3°	83.5°	81.5°	81.5°
**ANB**	3° ± 2°	−2.5°	0°	−0.5°
**Vertical Skeletal Relationships**				
**Maxillary inclination** **(S-N/ANS-PNS)**	8° ± 3°	8°	8°	8°
**MMPA**	27° ± 3°	28°	31°	30°
**LFH %**	55% ± 2%	58.5%	58.7%	58.6%
**Dento-Basal Relationships**				
**Upper incisor to Max. plane**	108° ± 6°	126.5°	126.5°	126°
**Lower incisor to Mand. Plane**	93° ± 6°	94°	92°	83°
**Lower incisor to A-Po line**	1 ± 1 mm	+12.5 mm	+8 mm	+5 mm
**Inter-incisal angle**	133° ± 10°	111°	110°	120°
**E-Line**				
**Upper lip-E line**	−2 ± 2 mm	−3 mm	−2 mm	−3 mm
**Lower lip-E line**	0 ± 2 mm	+4 mm	+3 mm	−1 mm

## Data Availability

Data are contained within the article.
